# Association of Coagulopathy and Inflammatory Biomarkers with Severity in SARS-CoV-2-Infected Individuals of the Al-Qunfudhah Region of Saudi Arabia

**DOI:** 10.3390/healthcare12070729

**Published:** 2024-03-27

**Authors:** Mohammad Asrar Izhari, Mansoor A. A. Hadadi, Raed A. Alharbi, Ahmed R. A. Gosady, Abdulmajeed Abdulghani A. Sindi, Daifallah M. M. Dardari, Foton E. Alotaibi, Faisal Klufah, Mohammad A Albanghali, Tahani H Alharbi

**Affiliations:** 1 Department of Laboratory Medicine, Faculty of Applied Medical Sciences, Al-Baha University, Al-Baha 65528, Saudi Arabia; 2 Laboratory Department, Qunfudhah Hospital, Al-Qunfudhah 28887, Saudi Arabia; 3 Laboratory Department, Baish General Hospital, Jazan 87597, Saudi Arabia; 4 Department of Basic Medical Sciences, Faculty of Applied Medical Sciences, Al-Baha University, Al-Baha 65528, Saudi Arabia; 5 Department of Genetic Counseling, Al-Faisal University, Riyadh 11533, Saudi Arabia; 6 Department of Public Health, Faculty of Applied Medical Sciences, Al-Baha University, Al-Baha 65528, Saudi Arabia

**Keywords:** regression, SARS-CoV-2, PTT, severity, CRP, odds ratio, D-dimer, ferritin, PT

## Abstract

Background: Identifying prognosticators/predictors of COVID-19 severity is the principal focus for early prediction and effective management of the disease in a time-bound and cost-effective manner. We aimed to evaluate COVID-19 severity-dependent alteration in inflammatory and coagulopathy biomarkers. Methods: A hospital-dependent retrospective observational study (total: n = 377; male, n = 213; and female, n = 164 participants) was undertaken. COVID-19 exposure was assessed by performing real-time PCR on nasopharyngeal (NP) swabs. Descriptive and inferential statistics were applied for both continuous and categorical variables using Rstudio-version-4.0.2. Pearson correlation and regression were executed with a cut-off of *p* < 0.05 for evaluating significance. Data representation by R-packages and ggplot2. Results: A significant variation in the mean ± SD (highly-sever (HS)/moderately severe (MS)) of CRP (HS/MS: 102.4 ± 22.9/21.3 ± 6.9, *p*-value < 0.001), D-dimer (HS/MS: 661.1 ± 80.6/348.7 ± 42.9, *p*-value < 0.001), and ferritin (HS/MS: 875.8 ± 126.8/593.4 ± 67.3, *p*-value < 0.001) were observed. Thrombocytopenia, high PT, and PTT exhibited an association with the HS individuals (*p* < 0.001). CRP was correlated with neutrophil (r = 0.77), ferritin (r = 0.74), and WBC (r = 0.8). D-dimer correlated with platelets (r = −0.82), PT (r = 0.22), and PTT (r = 0.37). The adjusted odds ratios (Ad-OR) of CRP, ferritin, D-dimer, platelet, PT, and PTT for HS compared to MS were 1.30 (95% CI −1.137, 1.50; *p* < 0.001), 1.048 (95% CI −1.03, 1.066; *p* < 0.001), 1.3 (95% CI −1.24, 1.49, *p* > 0.05), −0.813 (95% CI −0.734, 0.899, *p* < 0.001), 1.347 (95% CI −1.15, 1.57, *p* < 0.001), and 1.234 (95% CI −1.16, 1.314, *p* < 0.001), respectively. Conclusion: SARS-CoV-2 caused alterations in vital laboratory parameters and raised ferritin, CRP, and D-dimer presented an association with disease severity at a significant level.

## 1. Introduction

Regional outbreaks caused by SARS-CoV-2-variants/sub-variants still constitute a challenge to the currently available vaccine and treatment options [1]. To implement precise diagnostic and therapeutic strategies, it is imperative to explore comprehensive clinical research on COVID-19 [2]. COVID-19 is an inflammatory condition, and therefore, the generation of a variety of acute-phase reactants is an expected phenomenon [3,4]. Circulating biomarkers that offer information on severe trauma, immune function, and acute inflammation may be indispensable for contemplating the disease’s progression and severe outcome [2,5]. Cytokine response storm with elevated inflammatory serological biomarkers and coagulopathy worsen outcomes in COVID-19-infected individuals [6,7]. The elevated levels of these crucial biomarkers have been associated with diseases with the worst prognoses as well as with the most severe COVID-19 symptoms leading to prolonged hospitalization and economic setbacks for healthcare facilities [8,9]. COVID-19 infections’ severity demonstrated association with elevated procalcitonin (PCT) levels [10], raised ferritin [11], increased erythrocyte sedimentation rate (ESR) [12], C-reactive protein (CRP) elevation [13], and interleukin-6 (IL-6) elevated concentration [14]. Delineating markers’ concentration in virus-infected individuals helps with diagnosis and risk stratification [15]. The CRP remains considerably elevated even before the appearance of crucial abnormalities on CT, which is the one that correlates most strongly with COVID-19 development [16,17]. High CRP levels are associated with thrombotic sequela of COVID-19 infection [18], comorbidity [19], and lymphopenia [20]. CRP has been recognized as the outcome predictor and discriminator of COVID-19 disease severity [21], and therefore its population-specific measurement encompasses the diagnostic/prognostic value for the disease, COVID-19, and may have a remarkable clinical impact [22]. Raised systemic ferritin level is an indicator of the acute phase and inflammatory response [23]. Hyperferritinemia due to excessive inflammatory response is associated with high mortality [24] and the need for direct therapeutic intervention to control inflammatory cascade [25]. Moreover, it is also associated with a poor recovery rate of infection [24,26,27]. Association of hyperferritinemia with mortality is reported; however, it is still not well understood that hyperferritinemia is a potential biomarker for evaluating COVID-19 progression [28]. It has often been a neglected prognostic tool for COVID-19 despite the affordability and availability of the ferritin measurement test [3]. Reports suggest that the COVID-19-allied death rate was associated with increased thrombosis tendency (hypercoagulability) and thromboembolism resulting in a thrombo-inflammatory state [29,30]. Fibrinolysis generates D-dimer as a degradation product of cross-linked fibrin. D-dimer (>1 μg/mL) level was associated with COVID-19-allied death probability in hospitalized patients. A 20-fold rise in the COVID-19-allied mortality risk with a D-dimer level of 1000 ng/mL was reported [31]. Although raised D-dimer concentration has been reported to demonstrate an association with the severe state in a few studies [24,32], however, whether the D-dimer is a potential prognosticator/predictor of the disease severity including coagulopathy remains to be fully comprehended. Coagulation profile, especially D-dimer, could be a possible tool to assess the thromboembolism in veins, coagulopathy, and severity in COVID-19-infected patients [33]. COVID-19 clinical outcome is dependent on intrinsically complicated interaction of the host factors, including genetic makeup, with the virulence factors of SARS-CoV-2 [34,35]. Moreover, the clinical phenotype of COVID-19 and its severity vary individual-to-individual in a population considerably [34], which is suggestive of the accomplishment of the population-based study to determine the appropriate prognosticators of COVID-19 severity, and the outcome of the study could be used for early evaluation of the disease’s severity for timely, effective clinical management.

To the best of our knowledge, in the Qunfudhah population, an association of these biomarkers with COVID-19 severity has been undertaken for the first time. Additionally, because of limited information and lack of heterogenic cohort studies and meta-analysis in Qunfudah, the diagnostic/prognostic suitability of coagulopathy markers, such as D-dimer as well as inflammatory biomarkers viz CRP and ferritin, in predicting disease COVID-19 severity has not been well determined yet in the population of Qunfudhah, Saudi Arabia. Thus, we aimed to contemplate the CRP, ferritin, and D-dimer values and to determine their association with COVID-19 severity in the Qunfudhah region. 

## 2. Methods 

### 2.1. Design of the Investigation and Target Study Population

Hospital-based study design (retrospective, cross-sectional) was undertaken and accomplished on the data retrieved from laboratory records of Al-Qunfdah Hospital, Qunfudhah, Saudi Arabia, from January 2020 to December 2022. The medical laboratory records of SARS-CoV-2-infected participants who satisfied the inclusion and exclusion standards (criteria) laid down for the current research were accessed, reviewed, and analyzed for data acquisition. The demographic parameters such as age and the gender of the participants were obtained after inquisitive examination of the records of the selected participants.

### 2.2. Ethical Approval Statement

The Declaration of Helsinki was followed for carrying out this research. This investigation received approval from the ethics board of Al-Qunfudah, Hospital, Qunfdah, Makkah, Saudi Arabia (Reference No. 605-44-0057116) dated 6 October 2022. The application of the collected data for scientific study was clearly explained to the ethics committee via the proposal submitted before the commencement of this research. Data confidentiality was maintained, and the approval issued for this research was as per the guidelines of the ethics board policy for data protection. 

### 2.3. Sample Size Computations

Using the statistical formula n = N/(1 + Ne^2^), the ideal sample size was evaluated considering a confidence interval (95% C.I) and 0.05 as a margin of error (e) [36]. A sample size of (n = 399) was determined to commence this study, given N = 205,188. Nevertheless, a total of n = 377 participants met the eligible criteria for inclusion in this research. 

### 2.4. Eligibility Criteria

SARS-CoV-2-infected male and female participants of different ages comprised the study subjects. Participants infected with bacterial infections (*H. pylori*) who were prolonged users of proton pump inhibitors, patients with hormonal abnormalities (calciotropic abnormalities), pregnant women, patients with chronic kidney diseases (CKD), hemodialysis (HD), or CVD, and transplant patients were excluded. Individuals who underwent surgery and suffered from inflammatory disease were not included in this study. 

### 2.5. Laboratory Investigation Technique and Primary Data Generation Strategies

**Figure 1:**
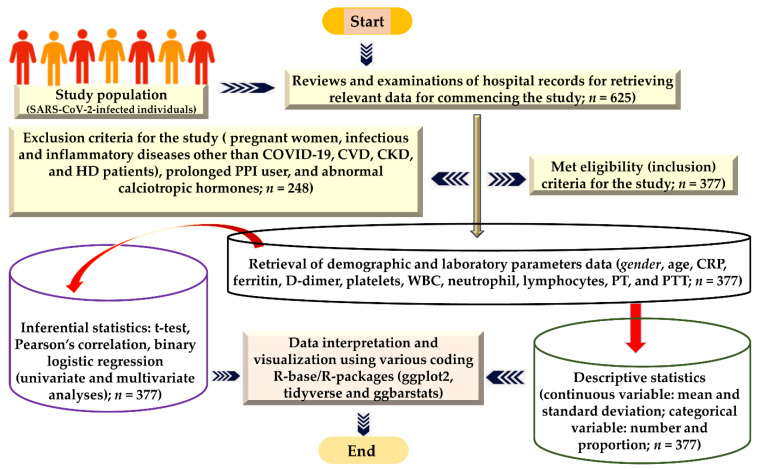
Strategies and resources employed for the execution, analysis, and illustration of the interpretation of findings.

Exposure assessment was executed by collecting throat swabs aseptically from individuals suspected to be infected for SARS-CoV-2 exposure examination and re-examination at a specific specimen collection center for COVID-19-suspected individuals at Qunfudhah Hospital. Complying with the manufacturer’s instructions, specimens were processed and analyzed by employing real-time reverse transcription polymerase chain reaction (RT-PCR). The viral-genomic RNA analysis was accomplished using an open reading frame 1b/nucleocapsid (ORF1b/N) gene PCR kit (KOGENEBIOTECH, Seoul, Republic of Korea) and previously reported standard operating procedures [37]. For assessing the hematological parameters of the COVID-19-positive individuals, patients’ fasting whole blood specimens were collected in tubes (EDTA-treated) and analyzed within 30 min of the collection time. Beckman Coulter DxH 900 was employed to determine the complete blood count (CBC). Viros System Integrated XT 7600 equipment was used to assess the laboratory parameter C-reactive protein (CRP). Ferritin level was determined by a DxI 800 analyzer (Beckman Coulter, Brea, CA, USA). The Stago automated device measured a light-blue tube (3.2% sodium citrate) containing D-dimer. The diagnosis and categorization of COVID-19 were according to the Saudi National Health Council’s COVID-19 recommendations and treatment (MOH 2020). Data obtained (demographic, laboratory parameter) and included in this study were reviewed multiple times, and a rigorous collection procedure was ensured to avoid collection errors. The severity status of COVID-19 disease (moderately severe and highly severe) was obtained from the hospital record based on the need for a ventilator, CT score, and ICU admission for highly severe patients, while the rest of the admitted patients with normal oxygen saturation were admitted to the COVID ward for moderately severe disease. Data were added to the MS Office/excel sheet for stratification and further processing. Incomplete/missing data were identified and removed manually to avoid discrepancies. A comprehensive self-explanatory illustration of the method adopted for carrying out this study is demonstrated in Figure 1.

### 2.6. Quality Assurance of the Data

All probable technical biases and errors were identified to be eliminated to ensure data quality. Multiple reviews were undertaken to remove incomplete data and data with discrepancies. 

### 2.7. Analytical Processes for Data Interpretation 

Data were stratified based on the gender of the participants into male and female categories. The whole set of data was also stratified into COVID-19 highly severe and moderately severe groups based on the severity status of the participant from their hospital records to compare the variation in the critical parameters under study. A total of n = 625 laboratory parameters were obtained from the laboratory records. Complete analytical methods (descriptive and inferential) were adopted to analyze the continuous and categorical variables using Rstudio-version-4.0.2. Continuous variables were summarized in terms of mean with standard deviation, interquartile range (IQR), and categorical variables were described as frequencies as well as proportions. Correlation (Pearson’s correlation) and regression analysis were conducted with a statistical significance cut-off laid down as *p* < 0.05 and a confidence interval of 95%. The *t*-test was used to compare the means of two groups of severity status with a statistical significance cut-off *p* < 0.05. The chi-square test was conducted to compare the difference in the proportion of the two groups. Univariate and multivariate analyses were accomplished for prediction. Pearson’s analysis was carried out to determine the correlation between different laboratory parameters of the two categories. Data representation was conducted by employing R-packages and ggplot2.

## 3. Results

### 3.1. Baseline Features of the Research Participants

Overall (n = 377), male (n = 213), and female (n = 164) participants were included to deduce the COVID-19 severity-dependent modulation in coagulation and inflammatory biomarkers. The descriptive statistical value for biomarkers such as CRP (mean ± SD: overall, 63.9 ± 44.0; male, 74.0 ± 43.1; female, 50.8 ± 41.8) in mg/L, D-dimer (mean ± SD: overall, 512.8 ± 169.4; male, 549.0 ± 168.5; female, 465.8 ± 159.1) in ng/mL, ferritin (mean ± SD: overall, 741.7 ± 174.7; male, 778.8 ± 181.6; female, 693.5 ± 152.8) in µg/L, thrombocytes/platelets (mean ± SD: overall, 211.1 ± 68.7; male, 197.9 ± 61.0; female, 228.2 ± 74.3) in 10^3^/µL, PT (mean ± SD: overall, 14.4 ± 3.9; male, 15.0 ± 4.9; female, 13.7 ± 1.6) in seconds, and PTT (mean ± SD: overall, 33.8 ± 7.0; male, 34.8 ± 7.1; female, 32.5 ± 6.5) in seconds were computed (Table 1). Participants’ baseline characteristics can be further comprehended by the mean ± SD for other hematological (laboratory) parameters and the median (IQR) for all the biomarkers in overall, male, and female categories, summarized in detail in Table 1. 

### 3.2. The Significant Difference in Coagulation-Disorder and Inflammatory Biomarkers by Severity Status

**Figure 2:**
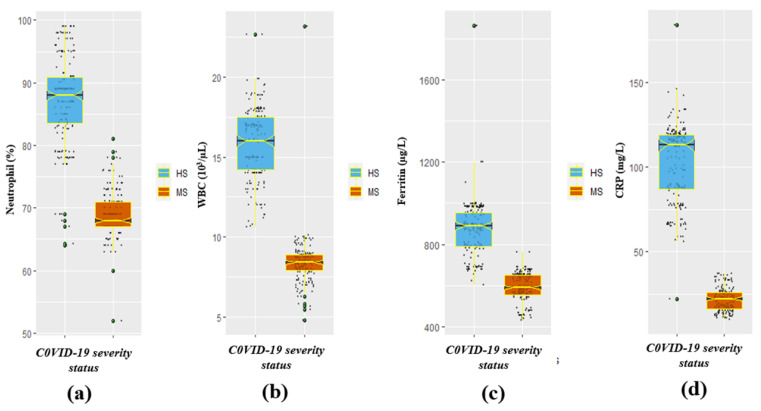
Illustration of vital inflammatory biomarkers stratified by COVID-19 disease severity: (**a**) neutrophil by severity, (**b**) WBC by severity, (**c**) ferritin by severity, and (**d**) CRP by severity status.

**Figure 3:**
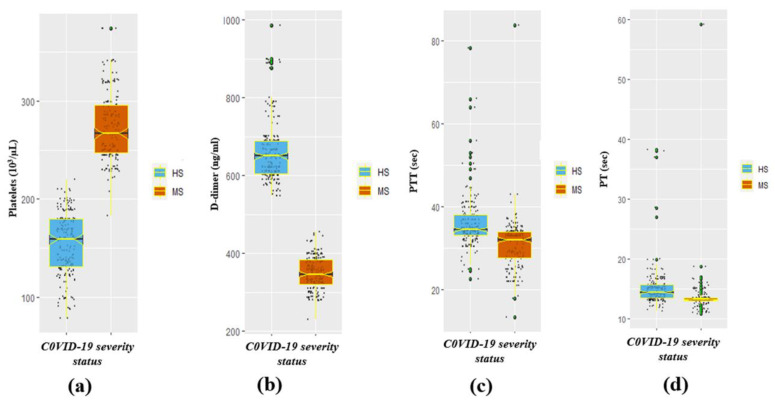
Representation of vital coagulation-disorder biomarkers stratified by COVID-19 disease severity. (**a**) Platelets by severity, (**b**) D-dimer by severity, (**c**) PTT by severity, and (**d**) PT by severity status.

A significant (*p* < 0.05) variation in the mean ± SD CRP (mean ± SD: highly severe (HS), 102.4 ± 22.9; moderately severe (MS), 21.3 ± 6.9; *p*-value < 0.001), D-dimer (mean ± SD: highly severe (HS), 661.1 ± 80.6; moderately severe (MS), 348.7 ± 42.9; *p*-value < 0.001), and ferritin (mean ± SD: highly severe (HS), 875.8 ± 126.8; moderately severe (MS), 593.4 ± 67.3; *p*-value < 0.001) of the HS and MS groups of the participants were observed (Table 2). The COVID-19-infected HS group exhibited significantly soaring concentrations of c-reactive protein (CRP), D-dimer, and ferritin compared to the MS category. Nevertheless, a significant (*p* < 0.05) drop in the concentration of platelets was observed in the HS category compared to the MS category (mean ± SD: HS, 154.0 ± 31.8; MS, 274.2 ± 34.9; *p* < 0.001), which is tabulated in Table 2. Moreover, the results also demonstrated a significantly (*p* < 0.05) increased prothrombin time (mean ± SD: HS, 15.3 ± 3.9; MS, 13.5 ± 3.6; *p*-value < 0.001) and partial prothrombin time (mean ± SD: HS, 15.3 ± 3.9; MS, 13.5 ± 3.6; *p* < 0.001) in the HS group in comparison to the MS category (Table 2). Furthermore, the other laboratory markers/parameters such as WBC, neutrophils, and lymphocytes in COVID-19 HS participants were also significantly high. The median (IQR) of neutrophil, WBC, ferritin, and CRP biomarkers by severity status is represented in Figure 2a–d respectively. Whereas the median (IQR) of platelets, D-dimer, PTT, and PT markers by COVID-19 severity status is illustrated in Figure 3a–d respectively. 

### 3.3. The Significant Difference in Coagulation-Disorder and Inflammatory Biomarkers by Gender

Gender-based analysis of the coagulation-disorder and inflammatory markers revealed significantly higher levels of CRP (mean ± SD: male, 74.0 ± 43.1; and female, 50.8 ± 41.8; *p*-value < 0.001), D-dimer (mean ± SD: male, 549.0 ± 168.5; and female, 465.8 ± 159.1; *p*-value < 0.001), and ferritin (mean ± SD: male, 778.8 ± 181.6; and female, 693.5 ± 152.8; *p* < 0.001) in males contrasted to females (Table 2). Additionally, gender-associated differences in the level of WBC counts, neutrophil percentage, PT, and PTT were significant, and males demonstrated higher levels compared to females (Table 2). 

### 3.4. COVID-19 Severity-Associated Modulation in the Coagulation-Disorder Markers and Inflammatory Biomarkers

**Figure 4:**
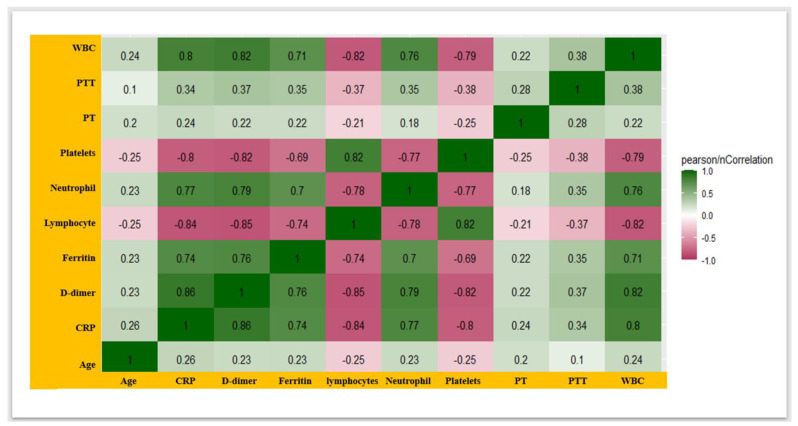
The correlogram represents the correlation matrix, which explains the polarity and strength of the correlation between several inflammatory and coagulation disorder biomarkers.

A comprehensive correlation matrix (correlogram) explaining the strength and polarity of the correlations between laboratory parameters in COVID-19-infected participants was deduced (Figure 4). The matrix demonstrated the positive correlations of CRP with neutrophil (correlation coefficient/cr = 0.77), ferritin (cr = 0.74), and WBC (cr = 0.8); nevertheless, the high-strength negative correlation of lymphocyte with ferritin (cr = 0.74) and CRP (cr = 0.84) was observed (Figure 4). Additionally, the matrix represented the correlation between vital coagulation-disorder laboratory parameters. D-dimer correlated with platelets (cr = −0.82), PT (cr = 0.22), and PTT (cr = 0.37), which is illustrated in Figure 4.

The adjusted odds ratio (Ad-OR) retrieved by regression analysis exhibited that for a unit enhancement in CRP value, the odds of being highly severe (HS) increase by 1.3 times (ad-OR: 1.30; C. interval: 1.137, 1.50; *p* < 0.001) contrasted to moderately severe (MS) individuals (Table 3). The odds of being HS were 1.048 times greater compared to MS with a unit increment in the ferritin level (ad-OR: 1.048; C. interval: 1.03, 1.066; *p* < 0.001). Other inflammatory indicators such as WBC (ad-OR: 4.612; C. interval: 2.98, 7.13) and neutrophil (ad-OR: 1.54; C. interval: 1.39, 1.68) were significantly (*p* < 0.001) associated with high disease severity (Table 3). Moreover, the odds of being highly severe compared to MS decreased by 84.7% (ad-OR: 0.153; C. interval: 0.015, 1.55; *p*-value = 0.112) with a unit increase in lymphocyte. Severity-associated changes in coagulation disorders were also assessed, and it was found that the odds of being HS increased by 30% (ad-OR: 1.3; C. interval: 1.24, 1.49; *p*-value > 0.05) compared to MS with one unit increment in D-dimer level (Table 3). Moreover, the odds of being HS was 34.7% and 23.4% greater compared to MS with a unit increment in the value of PT (ad-OR: 1.347; C. interval: 1.15, 1.57; *p*-value < 0.001) and PTT (ad-OR: 1.234; C. interval: 1.16, 1.314; *p*-value < 0.001), as summarized in Table 3. On the other hand, the odds of being HS decreased by 18.7% compared to MS with a unit increase in platelets (ad-OR: 0.813; C. interval: 0.734, 0.899; *p* < 0.001), as tabulated in Table 3.

## 4. Discussion

The heightened inflammatory response is a possible mechanism involved in COVID-19-associated-coagulopathy (CAC), which is one of the crucial complications of the SARS-CoV-2 infection and disease progression [35]. Additionally, massive inflammation-mediated respiratory distress, CAC’s manifestation as coagulation disorders (micro/macro-thrombi), may lead to multiple organ (kidney, brain, heart) damage in addition to damage to the lungs during disease progression has been reported. Several pieces of evidence suggest that apart from imaging methods, cytokine profiling is an invaluable tool for delineating the severity of the disease [38]; however, CRP, ferritin (inflammatory markers), and D-dimer (coagulation biomarkers) may serve as highly inexpensive and advantageous predictors/prognosticators of severity, especially, in low-medical-resource settings such as Qunfudhah [39,40,41]. Early assessment of the risk of disease severity and progression concerns the clinician [42], which necessitates the development of effective management strategies to prevent multiple organ damage and mortality outcomes; therefore, in this study, the determination of prognosticator/predictor (inflammatory and coagulation markers) of disease severity with great clinical value has been accomplished.

The predictive value of CRP for COVID-19 severity has been highlighted in many previous studies [43] along with other laboratory parameters, especially using machine learning and neural networking approaches for accurate prediction and deduction of decision trees [44,45,46]. In addition, the association of CRP with the most mutated and highly transmissible Omicron variants of SARAS-CoV-2 has also been reported [47]. Elevated CRP (mean ± SD: HS/MS, 102.4 ± 22.9/21.3 ± 6.9), and ferritin (mean ± SD: HS/MS, 875.8 ± 126.8/593.4 ± 67.3) were significantly (*p*-value < 0.001) associated with higher severity in the Qufudhah population, which was in line with other studies [12,40,41,42,43,44,45,46,47]. CRP (ad-OR: 1.30; 95%CI: 1.137, 1.50) and ferritin (ad-OR: 1.048; 95%CI: 1.03, 1.066) were significantly (*p* < 0.001) associated with HS (Table 3). Elevated CRP-associated mortality risk (11% increment in mortality for every 10 mg/L increase in CRP) has been reported in the previous study [48]. Additionally, reports also suggest severe diseases and higher mortality risk in individuals with elevated CRP and D-dimer [49,50]. In addition to that, elevated CRP was significantly associated with non-severe-to-severe aggravation of COVID-19, and the risk of advancing to the severe condition increased by five percent with a unit increase in CRP [51]. Furthermore, Mortoglu et al. showed the association of the level of CRP as well as other blood parameters with seasonal variation, which adds a new dimension to the study [52]. Previous studies suggest that for every 10 mg/L increase in CRP value, patients’ mortality increases by eleven percent [49]. Tahery et al. deduced the association of CRP with COVID-19 severity and mortality and suggested that CRP could be used as a potential biomarker for COVID-19 severity and fatality [53]. Moreover, Tahir Huyut et al. reported an abnormal level of inflammatory markers in patients who died of severe COVID-19 [54]. These coagulation-disorder and inflammatory markers could be used for early measurement of the oxidant/antioxidant level in COVID-19 severe cases by a model described by Huyut et al. [55] that is crucial for the effective management of the patients. In addition, Huyut et al. reported a laboratory biomarker-based machine learning model to predict COVID mortality [56]. Furthermore, potential markers could be used for the diagnosis of COVID-19 disease using machine learning-based sensors reported by Velichko et al. [57].

Poor inflammatory cascade management followed by acute respiratory distress syndrome (ARDS) constitutes a greater death rate probability [58], which supports the significance of CRP as a promising predictor of severe presentation in the early stage of the disease, especially in low-resource healthcare facilities. Huyut et al. found CRP as the most effective routine blood parameter (RBP) as a COVID-19 prognosticator [59]. 

Several reports suggested the disease severity-associated increase in ferritin levels, including an investigation into autopsies of SARS-CoV-2-infected cases [60,61]. Enhanced ferritin was also reported in severe-through-critical events compared to moderate-through-mild cases [8], which corroborated the observations of the current study. Evidence suggests that the higher ferritin level was significantly allied with the increased ferritin concentration in HS compared to MS, complicated in comparison to uncomplicated, and intensive-care-unit (ICU) through non-ICU events/cases [3,23]. In addition, high in-hospital death rates and severe pneumonia were significantly associated with ferritin higher than a thousand units [40]. A recent report suggests that elevated ferritin was associated with the serious neuropsychiatric post-COVID-19 complication, brain fog, which reflects the chronic inflammatory-mediated development of brain fog [62]. Hyperferritinemia could lead to ferroptosis and facilitate fibrin polymerization to induce procoagulant conditions [8]. Additionally, hyperferritinemia also contributes to the large-scale generation of reactive oxygen species (ROS) and renders oxidative stress, which eventuates in tissue toxicity and disease severity [23]. A few reports suggest the pathogenic potential of ferritin involved in inflammatory mechanisms as it triggers the expression of proinflammatory mediations and the release of cytokines from macrophages that contribute to disease progression and severity [63]. Thus, the evidence suggests the possible potential role of ferritin as an invaluable prognosticator of COVID-19 severity and clinical outcome.

The level of coagulation biomarker D-dimer (mean ± SD: HS/MS, 661.1 ± 80.6/348.7 ± 42.9, *p* < 0.001; ad-OR: 1.3; C. interval: 1.24, 1.49; *p*-value > 0.05 for HS compared to MS) (Table 3) was observed in this research, which was in corroboration with the observations of previous investigations [64,65,66,67]. Our observations suggest that HS COVID-19 patients were at greater risk of progressing to hypercoagulability (increased coagulation), which is supported by the findings of several other studies [68,69]. Enhanced D-dimer is significantly associated with non-surviving patients, reflecting the possible role of the D-dimer as a prognosticator/predictor of enhanced coagulability and thrombotic risk [30,70]. Moreover, our findings of raised PT, D-dimer, and PTT in HS participants were in line with a recent report that is reflective of the association of COVID-19-allied coagulopathy with increased PT, PTT, and D-dimer [71]. Based on the previous and current research’s findings, PT, D-dimer, and PTT should be taken into consideration while assessing the coagulation disorders in SARS-CoV-2-infected patients. Furthermore, higher males’ CRP (mean ± SD: male/female; 778.8 ± 181.6/693.5 ± 152.8), ferritin level (mean ± SD: male/female: 778.8 ± 181.6/693.5 ± 152.8), PT (mean ± SD: male/female; male, 15.0 ± 4.9/13.7 ± 1.6), and PTT (mean ± SD: male/female, 34.8 ± 7.1/32.5 ± 6.5) (Table 1) compared to females’ in SARS-CoV-2-infected individuals observed in the current study corroborated with previous findings [34,36,37,38]; on the other hand, lower males’ D-dimer (mean ± SD: male/female; 549.0 ± 168.5/465.8 ± 159.1) and thrombocytes (mean ± SD: male/female; 197.9 ± 61.0/228.2 ± 74.3) were inconsistent with findings of the gender association with D-dimer and platelets, probably due demographic difference [39]. Moreover, Mertoglu et al. reported an increased level of both inflammatory (CRP and ferritin) and coagulation-disorder biomarkers (D-dimer) in ICU patients compared to non-ICU patients, which reflects the association of disease severity with these markers [72]. The levels of ferritin, CRP, and D-dimer have been reported to be 1.75, 10.7, and 2.4 times higher in COVID-19 non-survivors [73].

Though the CRP and ferritin concentration is reflective of the measure (degree) of acute inflammatory response, however, lack of adequate information on the contribution of systemic inflammation in COVID-19 pathogenesis could be highlighted as future research avenues. Finding out highly necessitated potential prognosticators/predictors of disease severity which could be easily available with applicability even in low-resource healthcare systems to prevent disease progression in a time-bound manner and to avoid life-threatening post-COVID-19 complications is recommended. The limitation of this study could be unmeasured confounders such as the use of steroid anti-inflammatory medicine (dose and duration) and tocilizumab (interleukin-6 inhibitors) which might have been taken by a few patients before measuring inflammatory and coagulation-disorder markers.

## 5. Conclusions

Identifying promising tools for early diagnosis and prediction of COVID-19 disease severity is paramount for time-bound appropriate therapy and effective clinical management of the disease’s progression along with post-COVID-19 complications, especially in low-resource healthcare settings. In addition to heightened inflammatory reactions-mediated injury, coagulopathy has been reported as one of the life-threatening COVID-19 complications that contribute to poor outcomes and enhanced mortality in SARS-CoV-2-infected participants. Previous reports suggest that COVID-19 severity varies even at the individual level presumably because of the different immune statuses of the individuals in a population, which is suggestive of studying COVID-19 severity predictors in different populations to identify the clinical significance of these severity biomarkers for that population. Additionally, the information on the association of COVID-19 disease severity with D-dimer and inflammatory biomarkers in this study population is lacking. Therefore, in this study, potential coagulopathy (D-dimer, PT, and PTT) and inflammatory biomarkers (CRP and ferritin) have been delineated as readily available and inexpensive prognosticators/predictors of COVID-19 severity in the Qunfudhah population which could be used for early prediction of severity and timely initiation of appropriate therapy to avoid severe complications. Contemplation of the role of ferritin, CRP, as well as D-dimer in SARS-CoV-2-triggered systemic inflammation, may be a future research avenue. Unmeasured confounders, such as the use of anti-inflammatory drugs and duration, could be highlighted as limitations of the present study.

## Figures and Tables

**Table 1:** Basic characteristics of the study participants (n = 377).

	Overall		Males		Females	
Characteristic	Mean ± SD N = 377	Median (IQR) N = 377	Mean ± SD N = 213	Median (IQR) N = 213	Mean ± SD N = 164	Median (IQR) N = 164
CRP (mg/L)	63.9 ± 44.0	65.0 (22.0, 113.0)	74.0 ± 43.1	84.0 (25.0, 115.0)	50.8 ± 41.8	26.5 (17.4, 88.0)
D-dimer (ng/mL)	512.8 ± 169.4	576.0 (350.0, 650.0)	549.0 ± 168.5	601.0 (380.0, 656.0)	465.8 ± 159.1	388.0 (333.8, 644.3)
WBC (10^3^/µL)	12.3 ± 4.2	12.0 (8.4, 16.1)	13.2 ± 4.1	14.0 (8.8, 16.6)	11.2 ± 4.1	8.9 (8.1, 15.3)
Neutrophil (%)	78.7 ± 10.8	78.0 (69.0, 89.0)	80.9 ± 10.5	83.0 (69.0, 89.0)	75.9 ± 10.5	71.0 (68.0, 86.0)
Lymphocyte (%)	16.6 ± 9.1	11.0 (8.5, 23.8)	14.6 ± 8.7	9.5 (8.3, 22.0)	19.1 ± 9.1	21.0 (8.7, 24.8)
Platelets (10^3^/µL)	211.1 ± 68.7	199.0 (152.0, 266.0)	197.9 ± 61.0	181.0 (150.0, 250.0)	228.2 ± 74.3	245.5 (161.0, 286.0)
Ferritin (µg/L)	741.7 ± 174.7	689.0 (594.0, 890.0)	778.8 ± 181.6	790.0 (634.0, 897.0)	693.5 ± 152.8	654.0 (576.8, 798.3)
Age (in years)	46.2 ± 23.4	46.0 (25.0, 66.0)	43.7 ± 22.8	44.0 (23.0, 62.0)	49.4 ± 23.9	50.0 (28.0, 67.0)
PT (sec)	14.4 ± 3.9	13.4 (13.0, 15.1)	15.0 ± 4.9	13.9 (13.1, 15.2)	13.7 ± 1.6	13.3 (13.0, 14.1)
PTT (sec)	33.8 ± 7.0	33.7 (31.0, 36.0)	34.8 ± 7.1	33.5 (31.8, 37.0)	32.5 ± 6.5	34.0 (29.0, 35.1)

CRP = C-reactive protein, WBC = white blood cells, PT = prothrombin time, PTT = partial prothrombin time, and sec = seconds.

**Table 2:** Characteristics of the participants by gender and the COVID-19 severity status.

	By Gender			By Severity Status		
Laboratory Profile	Female N = 164 ^1^	Male N = 213 ^1^	*p*-Value ^2^	HS N = 198 ^1^	MS N = 179 ^1^	*p*-Value ^2^
CRP (mg/L)	50.8 ± 41.8	74.0 ± 43.1	<0.001	102.4 ± 22.9	21.3 ± 6.9	<0.001
D-dimer (ng/mL)	465.8 ± 159.1	549.0 ± 168.5	<0.001	661.1 ± 80.6	348.7 ± 42.9	<0.001
WBC (10^3^/µL)	11.2 ± 4.1	13.2 ± 4.1	<0.001	15.9 ± 2.2	8.4 ± 1.5	<0.001
Neutrophil (%)	75.9 ± 10.5	80.9 ± 10.5	<0.001	87.4 ± 7.2	69.1 ± 3.6	<0.001
Lymphocyte (%)	19.1 ± 9.1	14.6 ± 8.7	<0.001	8.6 ± 1.5	25.4 ± 4.8	<0.001
Platelets (10^3^/µL)	228.2 ± 74.3	197.9 ± 61.0	<0.001	154.0 ± 31.8	274.2 ± 34.9	<0.001
Ferritin (µg/L)	693.5 ± 152.8	778.8 ± 181.6	<0.001	875.8 ± 126.8	593.4 ± 67.3	<0.001
Age (in years)	49.4 ± 23.9	43.7 ± 22.8	0.021	51.5 ± 24.5	40.3 ± 20.7	>0.05
PT (sec)	13.7 ± 1.6	15.0 ± 4.9	<0.001	15.3 ± 3.9	13.5 ± 3.6	<0.001
PTT (sec)	32.5 ± 6.5	34.8 ± 7.1	0.001	36.4 ± 6.5	31.0 ± 6.3	<0.001

^1^ Mean ± SD; ^2^ Welch two-sample *t*-test, HS = highly severe, and MS = moderately severe.

**Table 3:** Organization of the magnitude of the association of COVID-19 severity with the laboratory profile of the participants (*n* = 377).

	Univariate Binary Logistic Regression			Multivariate Binary Logistic Regression		
Characteristic	COR ^2^	95% CI ^1^	*p*-Value	AOR ^1^	95% CI ^1^	*p*-Value
CRP (mg/L)	1.295	1.14, 1.5	<0.001	1.308	1.137, 1.50	<0.001
D-dimer(ng/mL)	1.40	1.26, 1.59	<0.05	1.3	1.24, 1.49	<0.05
WBC (10^3^/µL)	4.745	3.1, 7.2	<0.001	4.612	2.98, 7.13	<0.001
Neutrophil (%)	1.55	1.41, 1.69	<0.001	1.54	1.39, 1.68	<0.001
Lymphocyte (%)	=0.1	0.014, 1.49	<0.001	0.153	0.015, 1.55	=0.112
Platelets (10^3^/µL)	0.823	0.76, 0.89	<0.001	0.813	0.734, 0.899	<0.001
Ferritin (µg/L)	1.05	1.032, 1.068	<0.001	1.048	1.03, 1.066	<0.001
PT (sec)	1.492	1.284, 1.73	<0.001	1.347	1.15, 1.57	<0.001
PTT (sec)	1.23	1.16, 1.3	<0.001	1.234	1.16, 1.314	<0.001

^1^ AOR = Adjusted Odds Ratio; 95%CI = 95% Confidence Interval. ^2^ COR = Crude/unadjusted Odds Ratio; AOR was obtained after adjustment for age and gender. The reference category for dependent variables was the moderately severe (MS) category.

## Data Availability

It can be obtained from the author in correspondence with appropriate-request.
